# Clinical Trials of Interferon Inhibitors in Systemic Lupus Erythematosus and Preliminary Real-World Efficacy of Anifrolumab

**DOI:** 10.31138/mjr.260624.cto

**Published:** 2024-06-30

**Authors:** Samuel Díaz-Planellas, Dimitrios Katsifis-Nezis, Antonis Fanouriakis

**Affiliations:** 1 Internal Medicine Department. “Gregorio Marañón” General University Hospital, Complutense University of Madrid, Madrid, Spain,; 2 Rheumatology Unit, Fourth Department of Propaedeutic Internal Medicine, National and Kapodistrian University of Athens, “Attikon” University Hospital, Athens, Greece

**Keywords:** systemic lupus erythematosus, interferon, anifrolumab

## Abstract

Approval of anifrolumab for the treatment of moderate-to-severe systemic lupus erythematosus (SLE) in 2021 marked the success of a long quest to target the interferon system, in a disease wherein the latter has long been considered to play a pivotal role. Prior to anifrolumab, a number of agents had been tested in early phase clinical trials in patients with SLE, with equivocal results. Following its approval and marketing in several countries, the first reports regarding efficacy and safety in real-life clinical settings have been published, which suggest remarkable efficacy in skin manifestations of the disease, even after prior failure to multiple immunosuppressive therapies. In this report, we provide a short overview of IFN inhibitors that have been used in clinical trials of SLE, with a focus on anifrolumab; we also review all available evidence to date regarding its real-world efficacy and safety.

## INTRODUCTION

Systemic lupus erythematosus (SLE) is a chronic autoimmune disorder which affects primarily young women of reproductive age, with a spectrum of manifestations that range from mild to organ- or life-threatening.^1^ Significant advances in our understanding of the etiopathogenesis of SLE has led to an expanding pipeline of therapies in several phases of development, targeting diverse components of the immune system which are thought to malfunction in SLE. Among the different facets of the immune system that participate in disease pathogenesis, dysregulation of the interferon (IFN) system has long been considered a major determinant, only paralleled by B-cell activation and autoantibody production.^2^ To underline this, SLE is often described as an interferonopathy,^3^ while cases of familial childhood-onset chilblain lupus are caused by mutations in IFN-inducible genes.^4,5^ A detailed overview on the role of type I interferons in SLE is provided in a separate, dedicated article of this issue (Drougkas K. et al, MJR 2024), and is beyond the scope of this article. Based on the above, type I IFNs have long been considered as attractive therapeutic targets for SLE. Although earlier studies did not lead to successful results, the endeavor ultimately led to the approval of anifrolumab, a selective type I IFN receptor inhibitor, following the large phase 3 TULIP trials. Following approval, the drug has been available in several countries, including Greece, with the first reports on its real-world efficacy being published.

In the present article, we provide a short overview of IFN inhibitors that have been used in clinical trials of SLE, with a focus on anifrolumab, and we also review all available evidence regarding real-world efficacy of the latter, following its release.

## CLINICAL TRIALS OF IFN INHIBITORS IN SLE, WITH A FOCUS ON ANIFROLUMAB

As stated, building on the understanding of the role of type I IFNs in SLE pathophysiology, several clinical trials have been conducted to evaluate therapies targeting this pathway. Studies of earlier drugs, prior to the success of anifrolumab, yielded inconsistent results, possibly reflecting the complexity of IFN physiology due to the numerous type I IFNs with overlapping functions.

### IFN-α kinoid

In a 36-week, phase IIb, randomised, double-blind, placebo-controlled trial, the efficacy and safety of the immunotherapeutic vaccine IFN-α kinoid (IFN-K) was evaluated in 185 patients with moderate to severe SLE and a positive IFN signature (based on a selection of 10 IFN-inducible genes).^6^ Although the study did not meet its primary endpoint of BICLA response rate, the drug induced neutralising anti-IFN-α2b antibodies in 91% of patients, thus significantly reducing the IFN gene signature. Additionally, a higher attainment of lupus low disease activity state (LLDAS), significant glucocorticoid sparing, and improved outcomes in patients with neutralising antibodies were observed. The safety profile of IFN-K was deemed acceptable.

### Rontalizumab

The ROSE trial investigated rontalizumab, a humanised IgG1 anti-IFN-α monoclonal antibody against all 12 IFN-α subtypes preventing signalling through the type I IFN receptor, in 238 patients with moderate-to-severe SLE.^7^ The primary (British Isles Lupus Disease Activity Group [BILAG]-2004) and secondary (SLE Responder Index, SRI) endpoints of this phase II trial were not met in the overall patient population, nor in those with a high IFN signature (measured by an IFN signature metric, based on gene expression). Paradoxically, an exploratory analysis revealed that treatment with rontalizumab was associated with improvements in disease activity, reduced flares, and decreased glucocorticoid use in patients with low IFN scores. Although the reasons for this observation have not been thoroughly explained, it should be interpreted with caution due to the very low number of patients in the IFN-low group (n=28). Nevertheless, it still highlights the importance of stratifying lupus patients based on their IFN signature.

### Sifalimumab

Sifalimumab, a monoclonal antibody that directly binds multiple subtypes of IFN-α, was tested in a multicentre phase IIb trial of 431 patients with active SLE who were randomised to one of three different dosages of sifalimumab or placebo, added to stable conventional background therapy.^8^ After 24 weeks, a greater percentage of patients on the drug reached the primary end point (SRI-4), compared to placebo (placebo: 45.4%; 200 mg: 58.3%; 600 mg: 56.5%; 1200 mg 59.8%). Interestingly, improvement was consistent across various measures, both global and organ-specific ones, like the Cutaneous LE Disease Area and Severity Index), however the drug was also associated with higher rates of herpes zoster infection. Further studies on sifalimumab were nevertheless halted, since the funding company prioritised anifrolumab, which showed superiority in a pre-phase III trial.

### Anifrolumab

Anifrolumab is a humanised IgG1k monoclonal antibody that binds the type I IFN receptor 1 (IFNAR1). Unlike monoclonal antibodies against only IFN-a, anifrolumab targets the receptor, thereby providing a total inhibition of all type I IFNs (that is, IFN-α, IFN-β, IFN-ε, IFN-κ, and IFN-ω).^9^ Three randomised controlled trials have tested its efficacy and safety in SLE.

Initially, the MUSE study was a multicentre randomised placebo-controlled phase IIb trial involving 305 SLE patients.^10^ The latter were randomised 1:1:1 based on their baseline IFN scores, disease activity and oral glucocorticoid dosage, to IV anifrolumab 300mg or 1000mg/month or placebo. The primary end point was the achievement of SRI-4 at week, 24 along with a reduction of glucocorticoid use. Secondary end points were measured at week 52. SRI-4 was achieved in 34.3% of patients receiving 300mg, 28.8% receiving 1000mg and 17.6% on the placebo, with a more significant effect observed in patients belonging to the high IFN subgroup. Improvements were also noted in the secondary end points (BICLA, CLASI, tender joints).

Following the successful phase II trial, the two phase III TULIP trials were launched simultaneously and had an identical design. TULIP-1 included 457 patients, aged 18–70 years, with moderate-to-severe lupus.^11^ Participants were randomised 2:1:2 to receive IV anifrolumab 300mg/month, 150mg/month, or placebo, respectively. Similar to MUSE, the primary end point was the achievement of SRI-4 response, this time at 52 weeks, comparing the 300mg dose to placebo. As a surprise, while secondary end points, including BICLA, tender points and CLASI showed significant improvements, the trial did not meet its primary end point; a similar proportion of patients between anifrolumab and placebo reached the SRI-4 response (65 [36%] of 180) and placebo (74 [40%] of 184; p=0·41).

Based on the observation of a favourable BICLA response in patients who received anifrolumab in TULIP-1, and prior to unblinding the results of TULIP-2, the funding company modified the prespecified primary outcome measure of the latter from SRI-4 to BICLA, following approval by the Food and Drug Administration of the United States. In TULIP-2, 365 participants had been enrolled and randomised to anifrolumab 300mg/month or placebo.^12^ A significantly higher BICLA response was achieved in patients who received anifrolumab (48% vs 32% in placebo, p<0.05). Similar trajectories in other measures were also observed (SRI-4, CLASI and glucocorticoid dose). Regarding individual organ domains, although both TULIP-1 and TULIP-2 indicated treatment benefit of tender and swollen joints counts, the difference reached statistical significance only in *post hoc* analysis of pooled data from the two trials, and specifically in the IFN high patient subgroup. Treatment effects were more significant for swollen joints, possibly suggesting that swollen joints are a more accurate indicator of true joint inflammation.^13^ Results were also significant for the mucocutaneous domain, with a 16% difference over placebo in pooled data from the two studies (54% vs. 38% for BILAG and 55% vs. 39% for SLEDAI-2K muco-cutaneous manifestations).

Anifrolumab has also been tested in a phase II RCT in 147 patients with lupus nephritis (LN), using two different regimens (basic regimen 300 mg/month; intensified regimen 900 mg × 3 months, 300 mg thereafter) in combination with MMF and GCs, versus placebo.^14^ The study did not meet its primary endpoint of proteinuria reduction in combined anifrolumab groups versus placebo, however exploratory analyses showed that this was attributed to increased drug clearance and suboptimal exposure to anifrolumab in patients receiving the basic regimen. More patients in the anifrolumab intensified regimen achieved complete remission (∼ 15% difference compared to placebo) and sustained GC reduction (∼20% difference); currently, the drug is being tested in a larger phase 3 trial in LN.

Similar to other IFN inhibitors, anifrolumab is associated with an increased risk of herpes zoster infection. Although most cases in the trials were mild or moderate, caution is needed for this particular type of infection in patients starting the drug.

## REAL-WORLD EVIDENCE WITH ANIFROLUMAB

We reviewed all available literature on the real-life use of anifrolumab in patients with SLE. PubMed was searched for English language publications, using the terms Anifrolumab AND Lupus AND (Real life OR case report OR response OR Efficacy). All case reports and case series were included, irrespective of the number of patients they included. From the first reports, it was evident that anifrolumab exhibits remarkable efficacy particularly in skin manifestations of SLE.

### Characteristics of patients who were treated with anifrolumab

In total, 137 patients were included in the retrieved publications, with a mean age of 41.1 years. A detailed presentation is shown in **Table 1**. Nearly all patients who received anifrolumab had a history of long-standing disease, and in the majority anifrolumab was started due to cutaneous lupus refractory to multiple prior treatments. Indeed, essentially most patients (86%) had cutaneous involvement, with 77% of them presenting systemic manifestations. Excepting the LOOPS registry,^35^ which included patients who failed to achieve lupus low activity disease state despite receiving the existing standard of care, patients who achieved it but experienced minor flares and patients who achieved it but started anifrolumab to taper GCs, we found no report wherein anifrolumab was initiated due to refractory disease in a different organ system.

**Table 1.** Summary of studies reporting on real-world efficacy of anifrolumab in patients with systemic lupus erythematosus.

**References**	**N**	**Age/Sex**	**Organ Involvement**	**CLE Subtype**	**Disease duration**	**Prior systemic treatment**	**Concomitant treatment on ANI**	**Type of response**	**GC tapering**	**Adverse effects**
Plüß^15^	1	30-year-old Female	Arthritis, renal involvement.	SCLE	13 years	AZA, BEL GC, HCQ, MMF	GC, HCQ, MMF	CLASI-A drop from 17 to 7 at week 8	Yes, from 5mg to 2mg prednisolone.	None
Blum^16^	3	Mean 35-year-old (22–51) 3 females.	Haematological (2), MSK (2), Pericarditis (1), Renal involvement (2),	DLE (3) SLE + CLE (3)	11.3 years (9–13)	AZA (1), BEL (2), Dapsone (1), HCQ (3), MMF (2), MTX (1), PDN (3), RTX (1),	AZA (1), GC (3), HCQ (3), MMF (2)	Cutaneous response in 3 of 3 patients. SLEDAI or CLASI-A NR.	Discontinued GC at follow-up	None
Shope^17^	1	43-year-old. Female	Uveitis, Secondary Sjögren’s syndrome	SCLE (Rowell syndrome)	NR	AZA, HCQ, IVIg, MMF, PDN, RTX,	GC	Cutaneous response. SLEDAI or CLASI-A NR	Taper in 3 of 3 patients.	None
Trentin^18^	2	31-year-old	Arthritis	DLE + CHLE	Long-standing (NS)	GC, HCQ, conventional and biological immunosuppressants (NS)	GC + MTX	CLASI -A drop from 20 to 3 at month 5. SLEDAI-2K drop from 13 to 3 at month 6	NR	Mild COVID-19
		59 -year-old	None	DLE	Long-standing (NS)	Conventional immunosuppressants (NS), CYC, docetaxel, doxorubicin, GC, HCQ, JAKi	None	CLASI -A drop from 24 to 5 at week 4. SLEDAI-2K drop from 4 to 2 at week 4.	NR	None
Trentin^19^	20	Median age 48-year-old (24-68) 18 females 2 males	Antiphospholipid syndrome (3), Dermatological (18), Haematological (10), MSK (20), Neuropsychiatric (2) Renal (6), Serositis (5), Sjögren syndrome (2)	CLE in 18 of 20 patients (subtype NS)	12 years (4–27)	NR	AZA (3), CsA (1), GC (18), HCQ (15), MMF (6), MTX (6).	CLASI-A drop from 7 (0–27) to 2 (0–17) at month 1 and to 0 (0–2) at month 6. SLEDAI-2K drop from 6 (1–18) to 1 (0–2) at month 6.	Prednisone daily dose drop from 7,5 (0–25) to 5 (0–10) at month 6.	One patient discontinued treatment after 2 infusions due to a lack of response (NS) Mild viral infection (8) VZV (1) Thromboembolism (1)
Schaeffer^20^	1	42-year-old	Arthralgia, Pleural involvement, Renal involvement.	ACLE + SLE	30 years	AZA, BEL, CsA, GC, HCQ, LEN, MMF, MTX, photopheresis, RTX, THAL	BEL + GC + HCQ LEN + MTX + RTX + THAL	Cutaneous response. SLEDAI or CLASI-A NR	NR	Paradoxical joint and muscle pain. Slight increase of CPK and CRP. Potential interaction between ANI and BEL.
Shaw^21^	7	Mean 17-year-old (14–20) 6 females 1 male	Neuropsychiatric involvement (1), Pericarditis (1), Renal involvement (1)	DLE (7) SLE + CLE (3)	NR	AZA (2), BEL (2), CYC (1), dapsone (1), GC (7), HCQ (7), IVIg (2), LEF (1), MMF (7), MTX (4), QC (1), RTX (2), THAL (1)	GC (4), HCQ (7), IVIg (1), MMF (7),	CLASI-A drop from 22 (7–36) to 1 (0–3) at last follow-up (month 3-9) SLEDAI-2K drop from 13.5 (4–24) to 4.2 (0–10) at last follow-up (month 3–9)	Tapered GC in 3 of 4 patients under treatment with them	Recurrent HSV-1 reactivation in 1 patient.
Shaw^22^	7	Mean age 35 years 7 females	NR	SLE + DLE (7)	NR	Abatacept (1), AZA (2), BEL (2), CYC (1), dapsone (1), GC (5), HCQ (6), IVIg (2), LEF (1), LEN (2), MMF (5), MTX (4), QC (1), RTX (3), THAL (2), tofacitinib (1)	Acitretin (1), GC (1), HCQ (6), IVIg (2), MMF (4), THAL (1)	SLEDAI-2K drop from 10 (4–12) to 4.8 (0–8) at month 3 No CLASI-A available	Not possible in 1 of 1 patient under treatment with GC	None
Kowalski^23^	6	Mean 48-year-old (37–66) 5 females 1 male	Haematological (1), MSK (3), Pericarditis (1), Renal (3), VTE (3).	DLE (4), CCLE (2) SLE + CLE (5)	14 years (2–26)	AZA (4), BEL (4), CQ (4), CsA (1), CYC (2), Dapsone (2), GC (6), HCQ (6), IVIg (1), MMF (5), MTX (5), RTX (2),	AZA (1), CQ (1), GC (2), HCQ (3), IVIg (1)	Near-complete disease activity in 6 of 6. SLEDAI or CLASI-A NR.	Taper in 6 of 6 patients 4 of 6 are no longer with GC.	HSV (1) Worsening lupus nephritis (1)
Carter^24^	7	Mean 46-year-old (33–64)	Haematological (2), MSK (3)	SCLE (1), DLE (5) SLE + CLE (4)	17 years (6–26)	AZA (5), BEL (2), CsA (1), CYC (2), Epratuzumab (1), Etanercept (3), GC, HCQ (6), Isotretinoin (2), MMF (5), MTX (5), RTX (6), THAL (4), Ustekinumab (3)	GC (4)	Mean CLASI-A drop from 17 (10–26) to 6 (0–13) at 1 month and at 0 (0–4) at 3 months	4 of 5 patients on background GC achieved taper to 5mg prednisolone daily or discontinuation.	URI (1), Bronchitis (1), Otitis externa (1), Moderate SARS-CoV-2 (1), VZV (1)
Viedma-Martínez^25^	1	19-year-old female	Lymphopenia	SLE + CHLE	Paediatric onset (NS)	Apremilast, AZA, colchicine, dapsone, doxycycline, GC, HCQ, JAKi, mepacrine, MTX, nifedipine, sulfasalazine	NR	CLASI-A drop from 33 to 1 at week 4. SLEDAI NR.	NR	None
Günther^26^	7	Mean 43-year-old (22–67) 5 females 2 males	NR	DLE (4), SCLE (2), ACLE (1), mucosal involvement NS (3)	NR	Baricitinib (1), BEL (3), GC (2), HCQ (6), MMF (3), MTX (2),	HCQ (6), MMF (2), MTX (2), GC (2)	CLASI-A baseline drop from 16 (10–35) to close to 0 at month 3.	Yes, in 2 of 2 patients.	Mild viral respiratory infection (1) VZV (1)
Merola^27^	1	52-year-old female	Arthralgias, Fatigue, Nasal ulcers, Xerostomia and Xerophthalmia	SCLE + SLE	NR	BEL, CQ, GC, HCQ, MMF, MTX	GC (2), HCQ (6), MMF (2), MTX (2)	Near-complete clearance of the lesions (No CLASI-A or SLEDAI NR)	Yes (tapered off)	None
Chasset^28^	11	Mean 35- year-old (19–50)	None	DLE (10), SCLE (4), CHLE (1)	12 years (3–23)	AZA (4), BEL (11), CQ (1), CYC (1), GC (10), HCQ (11), MMF (4), MTX (8), RTX (3), THAL or LEN (11),	CQ (1), GC (9), HCQ (10), MMF (2), MTX (2), THAL or LEN (2)	Mean CLASI-A drop from 15 (4–35) to 4 at week 4 (0–19) and 2 (0–13) at week 16. Mean SLEDAI drop from 8 (4–22) to 4 (0–10) at week 16.	Median dose of prednisone decreased from 10mg/day (0–15) to 5mg/day (0–10)	Non-severe COVID-19 (2), VZV (1), Worsening preexisting palmar warts (1), Mucosal candidiasis (1), Hepatic cytolysis (1), Blurred vision/diplopia (1), Asthenia (2), Isolated increased CRP levels (1) No patient discontinued anifrolumab because of an AE.
Martín-Torregosa^29^	3	Mean 38-year-old (36–47) Female 3/3	MSK (3), Secondary Sjögren (1)	SCLE (3), CHLE (2) LP (1) SLE + CLE (3)	NR	BEL (1), GC (3), HCQ (3), LEF (1), Mepacrine (1), MMF (1), MMF (2), MTX (3)	MMF (1), MTX (1)	Mean CLASI-A drop from 30 (18–43) to 0 at week 16	No GC associated to ANI.	None
Woodbury^30^	2	35-year-old female	Arthritis, APS antibody syndrome, Autoimmune haemolytic anaemia, Autoimmune hepatitis	CHLE + DLE	NR	ASA, AZA, BEL, botulinum toxin, CQ, Dapsone, DXZ, HCQ, JAKi, LEN, MMF, MTX, RTX, Sildenafil, topical ruxolitinib.	ASA + MMF + RTX	CHLE and arthritis response. SLEDAI or CLASI-A NR.	No GC associated to ANI.	None
		48-year-old female	Sjögren syndrome SLE (NS)	CHLE	NR	ASA, BEL, CQ, GC, HCQ, LEN, MMF, MTX, RTX, THAL.	ASA + CQ + RTX + MTX	CHLE response. SLEDAI or CLASI-A NR.		None
Han^31^	1	21-year-old	None	DLE	3 years	AZA, GC, HCQ, topic tacrolimus.	GC + HCQ + topical treatment	Cutaneous response and hair regrowth. No CLASI-A available	No GC associated to ANI.	None
Khan^32^	2	39-year-old	Enteritis, leukopenia, Renal involvement	SCLE	18 years	BEL, Corticotropin, GC.	GC + HCQ + MMF	Cutaneous and haematological response. CLASI-A or SLEDAI NR	NR	None
		28-year-old	Fatigue, Polyarthralgia.	SLE + SCLE	1 year	BEL, Dapsone, GC, HCQ, MMF	Dapsone + GC + HCQ + MMF	Cutaneous response. CLASI-A or SLEDAI NR	Yes, but not discontinued.	None
Bao^33^	4	Mean 46-year-old. 3 females 1 male	MSK (2), Haematological (4)	DLE (2) CHLE (1) Lupus tumidus (1) SCLE (1) SLE + CLE (4)	17 years in one of them. In the other 3 patients NR.	AZA (1), BEL (1), CQ (2), dapsone (2), dipyridamole (1), GC (4), HCQ (4), MMF (2), MTX (1), sodium thiosulfate (1), tacrolimus (2),	CQ (1), GC (3) HCQ (4), MMF (3)	Mean CLASI-A drop from 16.75 (3–40) to 3 (0–8)	Reported in 2 of the 3 patients with GC. In the other one NR.	One presumed anifrolumab-related immunologic response to calcinosis cutis. Myalgia and malaise after each infusion in another patient, with worsening proteinuria after the first one (confirmed class V LN). Same patient had URI after the fourth dose.
Paolino^34^	4	Mean 35-year-old (25–44).	Haematological (2), MSK (4), Myocarditis (1), Neurological (1), Renal involvement (3)	SLE with mucocutaneous involvement NS (3), DLE (1)	9 years (5–12)	AZA (1), Baricitinib (1), BEL (3), CsA (1), CYC (2), Evobrutinib (1), GC (4), HCQ (4), MMF (4), MTX (1), Topical pimecrolimus (1)	GC (3), HCQ (4), MMF (4)	Mean CLASI-A drop from 19.5 (12–27) to 12 (0–20) at week 4 and to 7.5 (4–24) at week 16.	All patients were able to low dose or discontinue.	Herpes labialis (1) Genital candidiasis (2) No patient discontinued anifrolumab
Miyazaki^35^	45	Mean 44-year-old. 41 females 3 males	General symptoms (33) Mucocutaneous (28) Neuropsychiatric (6) MSK (30) Cardiorespiratory (1) Renal (19) Haematological (14)	NS	11,6 years	NS	AZA (1), BEL (18), HCQ (38), MMF (6), MTX (3), RTX (1), Tacrolimus (1)	Mean SLEDAI drop from 4.9 to 2.5 at week 12. 5/8 patients with cytopenia who failed to achieve LLDAS showed improvement. Control of disease activity in neuropsychiatric SLE. Improvement in patients with minor flares without GC dose increase.	Mean glucocorticoid dose were tapered from 11.3mg to 7mg at week 12.	3 patients discontinued anifrolumab due to lack of response 1 patient discontinued due to allergic reaction VZV (1) Respiratory infection (7) Cystitis (1)
Karagenova^36^	1	69-year-old Female	Arthritis, Pleural involvement and LN class V	DLE	More than 30 years.	AZA, GC, HCQ, JAKi, MMF, MTX, Voclosporin.	GC, voclosporin.	Articular response with renal involvement under control, without further proteinuria.	GC tapered off and no further triamcinolone injections were required	Cystitis.

ACLE: acute cutaneous lupus erythematosus; ANI: anifrolumab; ASA: acetylsalicylic acid; AZA: azathioprine; BEL: belimumab CHLE: child blain lupus erythematosus CLASI-A: Cutaneous lupus Erythematosus Disease Area and Severity Index; CLE: chronic lupus erythematosus; COVID-19: Coronavirus disease 2019; CQ; chloroquine; CRP: C-reactive protein; CsA: cyclosporin A; CYC: Cyclophosphamide; DLE: discoid lupus erythematosus; DXZ: doxazosin; GC: glucocorticoids; HCQ: hydroxychloroquine; IVIg: intravenous immunoglobulins; JAKi: JAK inhibitors; LEF: leflunomide; LEN: Lenalidomide; LLDAS: lupus low disease activity state; LP: lupus panniculitis; MMF: mycophenolate mofetil; MSK: musculoskeletal; MTX: methotrexate; NR: not reported; NS: not specified; RTX: rituximab; SLE: Systemic Lupus Erythematosus; SLEDAI: Systemic Lupus Erythematosus Disease Activity Index; THAL: thalidomide; URI: upper respiratory infection; VTE: venous thromboembolism; VZV: varicella zoster virus.

Regarding specific skin manifestations, 36% of patients had chronic cutaneous lupus (of whom, discoid lupus in 95%) and 12.5% had SCLE, while acute cutaneous lupus was reported in only 2% of patients. Subtype of cutaneous lupus was not specified in 35% of patients.

### Real-life efficacy of anifrolumab in SLE

All patients in the retrieved publications presented a favourable response in skin manifestations following treatment with anifrolumab, which was evident from week 4 and maximised around 6 months after treatment initiation. Improvement was reported in all forms of recalcitrant cutaneous lupus, regardless of subtype, with almost complete resolution of the lesions in a large part of the treated patients. Although some of articles did not measure the clinical improvement in terms of CLASI-A, those who did quantify the improvement reported a clinically significant response, with decrease in CLASI-A in all articles (see **Table 1** for details). Only four patients showed no response of disease with anifrolumab.^19,35^ Most patients who were receiving systemic glucocorticoid therapy at the beginning of anifrolumab were able to taper dose at last follow-up. Our preliminary experience in 18 patients with cutaneous disease that had proven recalcitrant to more than 5 immunosuppressive agents also support robust efficacy of the drug in skin manifestations of SLE.^36^

In contrast to skin disease, descriptions of responses in other organ systems are very limited in the retrieved articles. Some reports have described improvement in musculoskeletal symptoms/arthritis^30,37^while a single article reported resolution of leukopenia following the initiation of anifrolumab.^32^

The LOOPS retrospective cohort study from Japan is the largest real-life study of anifrolumab to-date (45 patients), which however did not focus on specific organ manifestations; rather, it aimed to assess drug survival at 26 weeks, reporting on rates of LLDAS and flares. It showed that anifrolumab reduces disease activity and GC doses in patients who formerly failed to achieve LLDAS and in those who experienced minor flares after achieving low disease activity, being at least as effective as intensification of standard therapy, without the need to increase the dose of GCs.^35^

Regarding safety, most patients did not experience adverse events related to the administration of anifrolumab (see **Table 1**). In those who presented any adverse event, the majority were mild viral respiratory infections,^18,19,24,26,28^ with five patients experiencing VZV reactivation.^19,24,26,28,35^ In one patient, the appearance of lupus panniculitis plaques in the lower limbs was reported as a possible immunological reaction to the calcinosis cutis that the patient previously had.^33^ In two patients, myalgia after the infusion was reported,^20,33^ one of them presenting worsening proteinuria with a biopsy compatible with class V lupus nephritis.^33^ In the other patient, an increase in CPK and CRP was observed along with muscle pain, as a possible result of the interaction between belimumab and anifrolumab.^20^ Another patient also presented worsening of lupus nephritis,^23^ albeit another study highlights the maintenance of remission of class V lupus nephritis after starting voclosporin and adding anifrolumab due to refractory skin and joint symptoms.^37^ One patient had a thromboembolic event^19^; however, neither the type of thromboembolism, nor the presence of other possible predisposing factors were specified.

## CONCLUSIONS

Although still preliminary, the first reports on real-world efficacy of anifrolumab in SLE are very encouraging, especially regarding the efficacy of the drug in cutaneous manifestations, which affect the majority of patients with SLE. This observation is particularly important, because to date treatment of manifestations other than LN is largely based on empirical grounds and often follows a ‘trial-and-error’ approach. In this regard, the profound efficacy of the drug in such a frequent organ manifestation with significant impact in patients’ quality of life is particularly important to guide therapeutic decisions, also because such remarkable efficacy has rarely been seen with drugs tested or even approved in SLE. A similar efficacy has been recently reported in a phase II study of the TYK2 inhibitor deucravacitinib in SLE; given that TYK2 mediates signalling from the type I IFN receptor, this provides further support to the fact that the IFN system is particularly important for skin disease in SLE, in light also of recent reports showing in situ IFN production from keratinocytes in lupus patients.^38,39^ *Post-hoc* analyses from the TULIP trials suggest a beneficial effect in additional organ domains, and this remains to be proven also in real-life studies, which are eagerly awaited.
